# Dynamics in HIV‐DNA levels over time in HIV controllers

**DOI:** 10.1002/jia2.25221

**Published:** 2019-01-10

**Authors:** Véronique Avettand‐Fenoel, Tatiana Bayan, Elise Gardiennet, Faroudy Boufassa, Pauline Lopez, Camille Lecuroux, Nicolas Noel, Pauline Trémeaux, Valérie Monceaux, Brigitte Autran, Laurence Meyer, Asier Saez‐Cirion, Olivier Lambotte, Christine Rouzioux, the CODEX ANRS Cohort Study Group, Jean‐Pierre Faller, Patricia Eglinger, Pascal Roblot, M David Plainchamp, Hugues Aumaître, Martine Malet, Christine Rouger, Gérard Rémy, Melle Kmiec Isabelle, Jean‐Luc Delassus, Alain Devidas, Eric Froguel, Sylvie Tassi, Philippe Genet, Juliette Gerbe, Olivier Patey, Richier Laurent, Marie‐Christine Drobacheff, Aurélie Proust, Helder Gil, Laurence Gérard, Eric Oksenhendler, Frédéric Lucht, Véronique Ronat, Michel Dupon, Hervé Dutronc, Séverine Le Puil, Jean‐Luc Schmit, Nathalie Decaux, Jean‐Michel Molina, Caroline Lascoux, Sylvie Parlier, Jean‐Pierre Bru, Gaëlle Clavere, Jean‐François Delfraissy, Cécile Goujard, Katia Bourdic, Jean‐François Bergmann, Maguy Parrinello, Gilles Pichancourt, Yves Welker, Alain Lafeuillade, Philip Gisèle, Christophe Rapp, Melle Lerondel, Pierre de Truchis, Huguette Berthe, Vincent Jeantils, Fatouma Mchangama, Daniel Vittecoq, Claudine Bolliot, Paul Henri Consigny, Fatima Touam, Gilles Pialoux, Sophie le Nagat, Olivier Bouchaud, Patricia Honoré, François Boué, Mariem Raho‐Moussa, Laurence Weiss, Lio Collias, Dominique Salmon‐Céron, Marie‐Pierre Pietri, Olivier Blétry, Dominique Bornarel, Emmanuel Mortier, Zeng Feng, Jean‐Daniel Lelièvre, Christine Katlama, Yasmine Dudoit, Anne Simon, Catherine Lupin, Pierre‐Marie Girard, Michèle Pauchard, Sylvie Abel, André Cabié, Pascale Fialaire, Jean‐Marie Chennebault, M Sami Rehaiem, Luc de Saint Martin, M Jean‐Charles Duthe, Philippe Morlat, Sabrina Caldato, Didier Neau, Séverine LE Puil, Pierre Weinbreck, Djamila Makhloufi, Florence Garnier, Isabelle Poizot‐Martin, Olivia Fauche, Alena Ivanova, Patrick Yeni, Sophie Matheron, Godard Cyndi, François Raffi, Hervé Hüe, Philippe Perré, Pierre Marie Roger, Aline Joulie, Éric Rosenthal, Christian Michelet, Faouzi Souala, Maja Ratajczak, Marialuisa Partisani, Patricia Fischer, Louis Bernard, Pascale Nau, Bruno Marchou, Florence Balsarin, Renaud Verdon, Philippe Feret, Christine Jacomet, Lionel Piroth, Sandrine Gohier, Pascale Leclercq, Agnés Meybeck, Raphaël Biekre, Thierry May, François Caron, Yasmine Debab, M David Theron, Patrick Miailhes, M Stanislas Ogoudjobi, Patrick Mercié, Marc Gatfosse, Martin Martinot, Anne Pachart, Patrice Poubeau, Agnès Uludag, Philippe Arsac, Lydia Bouaraba, Isabelle De Lacroix Szmania, M Laurent Richier, Vincent Daneluzzi, Elisabeth Rouveix, Geneviève Beck‐Wirth, Philippe Romand, Laurent Blum, Martine Deschaud, Christophe Michau, Christian Bernard, Florence Salaun, Philippe Muller, Yves Poinsignon, Annie Lepretre, Martine Deschaud, Thierry Lambert, Laurent Hocqueloux, Patrick Philibert, Mame Penda Sow, Albert Sotto, Jean‐Paul Viard, Agnés Cros, Marc De Lavaissiere, Pascale Perfezou, M Jean Charles Duthe, Catherine Gaud, Mathilde Aurore Niault, Virginie Mouton‐Rioux, Jean–Philippe Talar, Dupont Mathilde, M Stéphane Natur, Hikombo Hitoto, M Ali Mahamadou Ibrahim

**Affiliations:** ^1^ Université Paris Descartes Sorbonne Paris Cité Paris France; ^2^ AP‐HP Laboratoire de Virologie CHU Necker‐Enfants Malades Paris France; ^3^ INSERM CESP U1018 Université Paris Sud Le Kremlin Bicêtre France; ^4^ INSERM UMR 1184 Immunologie des Maladies Virales et Autoimmunes (IMVA) Université Paris Sud Le Kremlin Bicêtre France; ^5^ CEA DSV/iMETI Division of Immuno‐Virology IDMIT Fontenay aux Roses France; ^6^ AP‐HP Laboratoire de Virologie CHU Cochin Paris France; ^7^ Institut Pasteur Unité HIV inflammation et persistance Paris France; ^8^ Cellular Immunology Laboratory Université Pierre and Marie Curie INSERM UMRS 945 Paris France; ^9^ AP‐HP, CHU Bicêtre Service de Médecine Interne et Immunologie Clinique Le Kremlin‐Bicêtre France; ^10^ Université Paris Sud UMR 1184 Le Kremlin‐Bicêtre France

**Keywords:** HIV reservoir, HIV controllers, total HIV‐DNA, dynamics, long‐term follow‐up

## Abstract

**Introduction:**

HIV controllers (HIC) maintain viraemia at low levels without antiretroviral treatment and have small HIV reservoirs. Nevertheless, they are heterogeneous regarding their risk of infection progression. The study of reservoirs can help elucidate this control. This study aimed to explore the factors implicated in the pathogenesis of HIV infection that are potentially associated with HIV reservoirs and their dynamics in HIC.

**Methods:**

Individuals living with HIV included in the ANRS‐CODEX cohort with at least two HIV‐DNA measurements between 2009 and 2016 were selected. The total HIV‐DNA levels had been quantified prospectively from blood samples. Mixed‐effect linear models estimated the HIV‐DNA dynamics over time.

**Results:**

The median (interquartile range (IQR)) HIV‐DNA level was 1.5 (1.3 to 1.9) log copies/million peripheral blood mononuclear cells at inclusion (n = 202 individuals). These low levels showed heterogeneity among HIC. Lower levels were then associated with the protective HLA‐B*27/B*57 alleles and/or lower HIV‐RNA level at inclusion, negative hepatitis C virus serology, lower HIV‐suppressive capacity of specific CD8 T cells and lower levels of immune activation and inflammation. Interestingly, mathematical modelling of the dynamics of HIV‐DNA over time (840 measurements) showed that the number of infected cells decreased in 46% of HIC (follow‐up: 47.6 months) and increased in 54% of HIC. A multivariate analysis indicated that HLA‐B*27/B*57 alleles, a low level of HIV‐RNA and a low level of HIV‐DNA at inclusion were markers independently associated with this decrease.

**Conclusions:**

These results offer new insights into the mechanisms of long‐term control in HIC. In half of HIC, the decrease in HIV‐DNA level could be linked to tighter viral control and progressive loss of infected cells. These findings allow the identification of HIC with a low risk of progression who may not need treatment.

## 1 Introduction

Human immunodeficiency virus type 1 (HIV‐1) controllers (HIC) are a rare group of individuals living with HIV who maintain HIV viraemia at extremely low or even undetectable levels in the absence of antiretroviral treatment 1. Nevertheless, they are a heterogeneous group composed of subsets with different characteristics; some of them experience immunologic and/or virologic progression 2, 3, whereas others have an extremely high level of control over infection for years 4. Several parameters have been associated with this spontaneous viral control (among others, protective human leucocyte antigen (HLA) alleles and effective HIV‐specific CD4 and CD8 T‐cell responses) 5, 6. The impact of those parameters on the control of HIV replication and on the evolution of HIV reservoirs determines the long‐term future of these HIC and raises the question of whether some or all of them need antiretroviral treatment.

The first studies on HIV‐1 reservoirs in HIC reported that compared with progressors, they had smaller HIV reservoirs in their blood 1, 7, 8, 9, 10, 11, 12. Investigating these reservoirs can help elucidate this long‐term control.

This study aimed to explore the factors involved in HIV pathogenesis (HIV‐RNA load, activation and inflammation biomarkers, the presence of allele HLA‐B*27 and/or ‐B*57 and specific immune responses) that could be associated with the magnitude and dynamics of blood HIV reservoirs in HIC. The large biobank of samples from the French national cohort of HIC (ANRS CO21 CODEX cohort) allowed the investigation of the HIV‐DNA dynamics in blood over the course of several years.

## 2 Methods

The French multicentre CODEX cohort (ANRS) included HIC based on the following characteristics: an individual living with HIV‐1 who never received antiretroviral treatment, with a follow‐up time longer than five years and with the last five HIV‐RNA plasma measurements lower than 400 copies/mL. In total, 222 HIC were included in this cohort and received annual follow‐up. HIC from this cohort with at least two measurements of HIV‐DNA between 2009 and 2016 were selected for this study. All patients gave a written informed consent. The study protocol was approved by the regional investigational review board (*Comité de Protection des Personnes Ile‐de‐France VII*, Paris, France) and performed in compliance with the tenets of the Declaration of Helsinki.

Total HIV‐DNA had been quantified prospectively in frozen peripheral blood mononuclear cells (PBMC) by an ultrasensitive method using the real‐time PCR GENERIC HIV‐DNA assay (Biocentric, Bandol, France), as described previously 4, 13. Two to six replicates per PCR were performed to analyse a large number of cells, and the threshold ranged from 3 to 83 copies/million PBMC depending on the available cell number. More than 90% of quantifications were performed with a threshold <20 copies/million PBMC.

Ultrasensitive HIV‐RNA quantifications (US HIV‐RNA) were performed using the Generic HIV real‐time PCR assay (Biocentric, Bandol, France) or an adaptation of the Roche Cobas Ampliprep/Cobas Taqman v2. The threshold ranged from 1 to 40 copies/mL, depending on the available plasma volume (0.5 to 15 mL). More than 90% of quantifications were performed with a threshold <5 copies/mL.

Cumulative HIV viraemia was calculated by summing the products of the log viral load and the time interval to the previous measurement over the entire period of follow‐up in the CODEX cohort.

Human leucocyte antigen typing was performed on PBMC with the complement‐dependent microlymphocytotoxic technique (InGen).

The activation of CD4 and CD8 T cells was analysed at inclusion as the surface expression of HLA‐DR and CD38 by flow cytometry on fresh whole blood for 111 HIC.

Interferon gamma‐induced protein 10 (IP‐10) was measured as a marker of inflammation in plasma from 58 HIC at inclusion with a FlowCytomix bead‐based multiplex immunoassay (eBioscience Inc., San Diego, CA, USA).

The HIV‐suppressive capacity of specific CD8+ T cells was measured in 199 HIC, as thoroughly described previously (log decrease in p24 production in cultures of CD4+ T cells infected *in vitro* when co‐culture in the presence of autologous CD8+ T cells) 14.

### 2.1 Statistical analysis

Baseline demographic and immunovirological characteristics at the time of enrolment were described by the median and interquartile range (IQR) or 95% confidence intervals (95% CI), when necessary, for continuous variables and percentages for discrete variables. Comparisons of qualitative variables were performed by using chi‐square or Fisher's exact tests, while comparisons of quantitative variables were performed with Student's *t*‐tests or Wilcoxon–Mann–Whitney tests. Mixed‐effect linear models (MELM) were used to estimate total HIV‐DNA dynamics over time. Predictors of the decrease in HIV‐DNA levels were identified by univariate and multivariate logistic regressions. The decrease in HIV‐DNA was defined in two manners. First, we defined the outcome as binary categories with the values of HIV‐DNA at enrolment below the median, which was considered as the main category of interest in the model, and the values of HIV‐DNA above the median as the referent group. Second, we then considered the variation in HIV‐DNA over time. We determined the slope over time of HIV–DNA for each HIC included in the study. We then categorized this slope into two categories of HIC, those who had a decrease in slope over time, which was the group of interest, and those who had an increase or a stable slope over time, which was the referent group. We tested each factor or marker significantly associated with these outcomes in two separate univariate models. The factors or markers significantly associated with a *p* ≤ 0.05 with each of these outcomes in univariate analysis were then included in the multivariate models. Values of *p* < 0.05 were considered significant.

HIV‐DNA and HIV‐RNA loads were set to the threshold when the markers were undetectable for statistical analysis. To evaluate the sensitivity and robustness of the results, the values of HIV‐DNA and HIV‐RNA below the threshold were set to a range of randomized values between 1 and the threshold. All these analyses gave similar results to those obtained with the threshold value.

## 3 Results

3In total, 202 HIV controllers were selected. The patient characteristics at inclusion are reported in Table 1; 50.5% were men, and 42% had protective HLA‐B*27 and/or HLA‐B*57 alleles (28 HLA‐B*27, 54 HLA‐B*57, 3 HLA‐B*27 and B*57). Patient characteristics at inclusion according to their status for the protective HLA‐B*27/B*57 alleles are presented in Table 2. The median (IQR) US HIV‐RNA load was 1.4 (0.6 to 2.1) log copies/mL at inclusion. US HIV‐RNA was undetectable in 68 HIC at inclusion. The median follow‐up after inclusion in the cohort was 47.6 months, IQR (26.2 to 61.8). During this follow‐up, 114 measurements of US HIV‐RNA from 73 HIC were undetectable among 838 measurements from the 202 HIC.

**Table 1:** HIV controller characteristics at inclusion in the ANRS CODEX cohort according to their protective B*27/B*57 HLA allele status

HIV controllers	Overall	HLA‐B*27/B*57 alleles (n = 85)	HLA non‐B*27/B*57 alleles (n = 117)	Comparison between HLA groups (p)
Men, n (%)	102 (50.5)	47 (55)	55 (47)	0.24
Age, median (IQR)	45.2 (39.2 to 51.2)	47 (41 to 54)	44 (38 to 50)	0.009
Transmission blood, n (%)	43 (21.3)	21 (25)	22 (19)	
Sex, n (%)	139 (68.8)	54 (64)	85 (73)	0.38
Other, n (%)	20 (9.9)	10 (11)	10 (8)	
HIV‐RNA (log copies/mL), median (IQR)	1.4 (0.6 to 2.1)	1.3 (0.6 to 2.1)	1.5 (0.6 to 2.1)	0.97
HIV‐DNA (log copies/million PBMC), median (IQR)	1.5 (1.3 to 1.9)	1.3 (1.3 to 1.9)	1.5 (1.3 to 1.9)	0.41
CD4 T‐cell count (cell/mm^3^), median (IQR)	765 (600 to 979)	775 (584 to 957)	762 (601 to 997)	0.55
CD4/CD8 ratio, median (IQR)	1.12 (0.77 to 1.58)	1.2 (0.7 to 1.6)	1.1 (0.8 to 1.6) (n = 115)	0.86
Expression of HLA‐DR and CD38 on CD4 T cells (%)	0.9 (0.4 to 1.4)	1.4 (1.1 to 2.0) (n = 35)	0.6 (0.4 to 1.1) (n = 76)	<0.001
Expression of HLA‐DR and CD38 on CD8 T cells (%)	2.7 (1.2 to 5.7)	4.7 (2.3 to 7.8) (n = 35)	2.2 (0.9 to 4.5) (n = 76)	0.002
Positive HCV serology, n (%)	22 (10.9)	13 (15)	9 (8)	0.09

HLA, human leucocyte antigen; HCV, hepatitis C virus; IQR, interquartile range.

**Table 2:** Factors associated with HIV‐DNA level <1.5 log copies/million PBMCs at inclusion in the CODEX cohort – univariate and multivariate analyses

	Univariate analysis		Multivariate analysis	
	Odds ratio (95% CI)	*p*‐value	Odds ratio (95% CI)	*p*‐value
Women	1.61 (0.93 to 2.81)	0.09	1.27 (0.68 to 2.37)	0.45
HLA‐B*27 and/or B*57	1.70 (0.97 to 2.99)	0.065	2.00 (1.07 to 3.76)	0.03
HIV‐RNA load at inclusion^a^	0.36 (0.24 to 0.55)	<0.001	0.37 (0.24 to 0.57)	<0.001
CD4 T‐cell count^b^	1.03 (0.95 to 1.12)	0.51	0.98 (0.89 to 1.07)	0.62
Positive HCV serology	0.26 (0.09 to 0.73)	0.01	0.25 (0.08 to 0.76)	0.02

HLA, human leucocyte antigen; HCV, hepatitis C virus; IQR, interquartile range; ORs, Odds ratios. ^a^ORs calculated for a 1‐log_10_ increase; ^b^ORs calculated for a 200‐cell increase.

### 3.1 Blood HIV‐DNA levels at inclusion

The HIV‐DNA levels were low (median (IQR): 32 (20 to 50) copies/million PBMC (1.5 (1.3 to 1.9) log copies/million PBMC)). Sixty HIC had undetectable levels of HIV‐DNA. Women had significantly lower HIV‐DNA levels than men 25 (95% CI 22 to 33) copies/million PBMC versus 42 (95% CI 26 to 59) copies/million PBMC ((1.40 (95% CI 1.34 to 1.52) log vs. 1.62 (95% CI 1.42 to 1.77) log; *p* = 0.02)) at inclusion in the cohort. There were no differences in HIV‐DNA levels according to transmission groups, sexual preference, transmission mode or ethnicity. The HIV‐DNA level was significantly higher in HIC with antibodies against the hepatitis C virus (HCV) (n = 22/202) than in HIC without antibodies against the HCV 74 (95% CI 47 to 155) copies/million PBMC versus 28 (95% CI 22 to 35) copies/million PBMC (1.87 (95% CI 1.67 to 2.19) log copies/million PBMC vs. 1.45 (95% CI 1.34 to 1.54) log, *p* = 0.005), although there was no difference in HIV‐RNA load at inclusion between those two groups (*p* = 0.25).

The HIV‐DNA level was significantly higher in HIC with HIV‐RNA ≥1 log copies/mL at inclusion (n = 125) than in HIC with HIV‐RNA <1 log copies/mL at inclusion 65 (95% CI 47 to 81) copies/million PBMC versus 22 (95% CI 20 to 24) copies/million PBMC (1.81 (95% CI 1.67 to 1.91) log copies/million PBMC vs. 1.34 (95% CI 1.30 to 1.38) log, *p* = 0.005). Moreover, it was also significantly higher in HIC with cumulative HIV viraemia above the median (3.48 log) during the follow‐up than in other HIC 48 (95% CI 34 to 65) copies/million PBMC versus 22 (95% CI 20 to 28) copies/million PBMC (1.68 (95% CI 1.53 to 1.81) log vs. 1.34 (95% CI 1.30 to 1.44) log, *p* < 0.001). None of the following factors (T‐cell activation (n = 111), HIV‐specific CD8 cell responses (n = 199) or IP‐10 (n = 58)) were found to be significantly associated with HIV‐DNA level < 1.5 log copies/million PBMCs (32 copies/million PBMC) at inclusion.

Overall, a univariate analysis of the entire group showed that an HIV‐DNA level <1.5 log (median value) was associated with a low HIV‐RNA level at inclusion and hepatitis C seronegativity. There was a borderline association between an HIV‐DNA level <1.5 log and the presence of HLA‐B*27 and/or B*57 alleles. Multivariate analysis showed that an HIV‐DNA level <1.5 log was significantly associated with a low HIV‐RNA level at inclusion, the presence of HLA‐B*27 and/or B*57 alleles and hepatitis C seronegativity (Table 2).

Data concerning activation and inflammation were available for a subgroup of HIC. Those with CD4 T‐cell activation as assessed by the coexpression of HLA‐DR and CD38 above the median value of 0.9% (n = 51) had significantly higher HIV‐DNA loads 50 (95% CI 25 to 81) copies/million PBMC versus 30 (95% CI 19 to 34) copies/million PBMC (1.70 (95% CI 1.40 to 1.91) log copies/million PBMC vs. 1.47 (95% CI 1.28 to 1.53) log for the other 60 HIC, *p* = 0.003). The 55 HIC with CD8 T‐cell activation as assessed by the coexpression of HLA‐DR and CD38 above the median value of 2.7% had significantly higher HIV‐DNA levels 50 (95% CI 25 to 74) copies/million PBMC versus 28 (95% CI 20 to 33) copies/million PBMC (1.70 (95% CI 1.39 to 1.87) log copies/million PBMC vs. 1.44 (95% CI 1.30 to 1.52) log in the other 56, *p* = 0.005), *p* = 0.005). The 82 HIC with CD4/CD8 ratios <1 also had significantly higher HIV‐DNA loads than the 118 HIC with ratios ≥1 (44 (95% CI 31 to 65) copies/million PBMC vs. 23 (95% CI 21 to 33) copies/million PBMC; 1.64 (95% CI 1.49 to 1.81) log copies/million PBMC vs. 1.36 (95% CI 1.33 to 1.52) log, *p* = 0.001). Noticeably, HIC with higher levels of CD8 T‐cell activation or CD4/CD8 ratios <1 had also significantly higher HIV‐RNA loads than other HIC (40 (95% CI 23 to 110) copies/million PBMC vs. 20 (95% CI 12 to 40) copies/million PBMC; 1.60 (95% CI 1.36 to 2.04) log vs. 1.3 (95% CI 1.08 to 1.60) log, *p* = 0.01, and 63 (95% CI 40 to 107) copies/million PBMC vs. 20 (95% CI 12 to 40) copies/million PBMC; 1.80 (95% CI 1.60 to 2.03) log vs. 1.30 (95% CI 1.08 to 1.60) log, *p* = 0.0004 respectively).

The HIV‐suppressive capacity of specific CD8 T cells was analysed for 176 HIC. Noticeably, those with CD8 T‐cell antiviral capacity above the median value (0.905 log p24 decrease; n = 88) had significantly higher levels of HIV‐DNA (34 (95% CI 25 to 51) copies/million PBMC vs. 23 (95% CI 20 to 36) copies/million PBMC; 1.53 (95% CI 1.39 to 1.71) log copies/million PBMC vs. 1.36 (95% CI 1.31 to 1.56) log for the other HIC, *p* = 0.02). These HIC with CD8 T‐cell antiviral capacity above the median value also had a cumulative HIV viraemia above the median (3.48 log) more frequently than HIC with lower CD8 T‐cell activities (n = 54 (61.4%) vs. n = 37 (42.1%), *p* = 0.01).

On the 58 subjects with available IP‐10, HIC with IP‐10 ≥ 50 pg/mL (median) tended to have higher levels of HIV‐DNA than those with IP‐10 < 50 pg/mL (1.60 log copies/million PBMC vs 1.34 log copies/million PBMC, *p* = 0.06), whereas no difference in HIV‐RNA load was observed between the two groups (*p* = 0.66).

### 3.2 Dynamics of HIV‐DNA levels in PBMC over years

**Figure 1:**
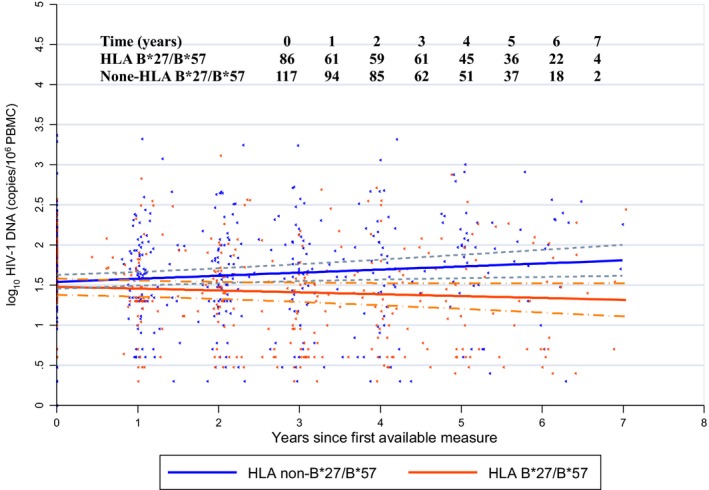
Model of the dynamics of the total HIV‐DNA levels, with 95% confidence intervals, in the blood of HIV controllers during a follow‐up period of more than six years, according to their HLA‐B*27 and/or B*57 status. Slope of HIV‐DNA load for HIC with protective HLA‐B*27 and/or B*57 alleles: −0.023 log copies/million PBMC/year; for HIC without HLA‐B*27 and/or B*57 alleles: +0.038 log copies/million PBMC/year, *p* = 0.002. Solid lines indicate the estimated means and dashed lines indicate the 95% confidence intervals around means log 10 DNA copies/million over time. HIC, HIV controllers.

In total, 840 measurements of HIV‐DNA were performed on sequential samples for the 202 HIC, with a median of five samples per individual (range 2 to 8). Among them, HIV‐DNA was undetectable in 181 measurements from 88 HIC. MELM showed that the slopes of HIV‐DNA loads over time were significantly different between HIC with protective HLA‐B*27 and/or B*57 alleles (−0.023 (95% CI −0.051 to +0.005) log copies/million PBMC per year, namely, −9 copies/million PBMC in six years) and HIC without HLA‐B*27 and/or B*57 alleles (+0.038 (95% CI +0.010 to +0.065) log copies/million PBMC per year, namely, +22 copies/million PBMC in six years, *p* = 0.002), the latter of whom experienced a small but significant increase in the level of HIV‐DNA over time (*p* = 0.006) (Figure 1).

**Figure 2:**
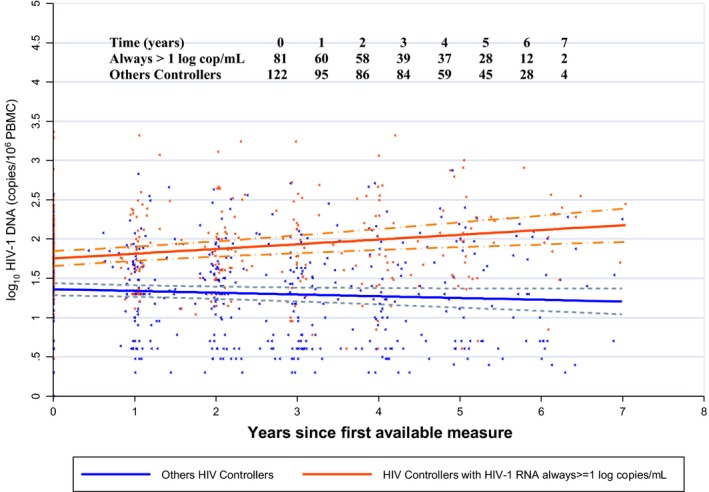
Model of the dynamics of the total HIV‐DNA levels, with 95% confidence intervals, in HIV controllers during a follow‐up period of more than six years, according to the levels of HIV‐RNA over time. Slope of HIV‐DNA load for HIC who always had HIV‐RNA ≥1 log copies/mL during follow‐up: +0.060 log/year; for other HIC: −0.022 log/year; *p* < 0.0001. Solid lines indicate the estimated means and dashed lines indicate the 95% confidence intervals around means log 10 DNA copies/million over time. HIC, HIV controllers.

The HIV‐DNA levels of HIC who always had HIV‐RNA ≥1 log copies/mL during follow‐up (n = 81) significantly increased over time (slope: +0.060 (95% CI +0.029 to +0.092) log/year, namely, +42 copies/million PBMC in six years, *p* < 0.0001) and differed significantly (*p* < 0.0001) from the slope of the HIV‐DNA levels of other HIC (slope: −0.022 (95% CI −0.046 to +0.001) log/year, namely, −31 copies/million PBMC in six years) (Figure 2).

There was no difference in HIV‐DNA slopes according to the HIV CD8 T‐cell response. None of the following factors (T‐cell activation, HIV‐specific CD8 cell responses or IP‐10) were found to be significantly associated with a decrease in HIV‐DNA over time.

Overall, HIC for whom HIV‐DNA load decreased (n = 93, 46%) were more often women (*p* = 0.025), were significantly more likely to have HLA‐B*27 and/or B*57 alleles (*p* = 0.001), had a significantly lower HIV‐RNA load at inclusion (median (95% CI): 0.60 (0.30 to 1.02) log copies/mL vs. 1.78 (1.70 to 2.01) log, *p* < 0.001) and had a significantly lower HIV‐DNA load at inclusion (median (95% CI): 1.30 (1.30 to 1.34) log copies/million PBMC vs. 1.76 (1.60 to 1.89) log, *p* < 0.001). The two groups had no difference in CD4 T‐cell counts at inclusion (median (IQR): 864 (792 to 933) vs. 787 (725 to 848) cells/mm^3^, *p* = 0.11). A multivariate analysis indicated that the presence of HLA‐B*27 and/or B*57 alleles, a low HIV‐RNA level at inclusion and a low HIV‐DNA level at inclusion were independently associated with the decrease in HIV‐DNA load over time (Table 3). Noticeably, HIC who experienced a decrease in HIV‐DNA level over time had a slope of HIV‐RNA load that was not different from 0 (*p* = 0.75), whereas HIC who experienced an increase in HIV‐DNA over time also experienced a significant increase in HIV‐RNA (+0.036 log copies/mL per year, *p* = 0.01).

**Table 3:** Factors associated with a decrease in HIV‐DNA over time in the CODEX cohort – univariate and multivariate analyses

	Univariate analysis		Multivariate analysis	
	Odds ratio (95% CI)	*p*‐value	Odds ratio (95% CI)	*p*‐value
Women	1.90 (1.08 to 3.32)	0.025	1.47 (0.73 to 2.96)	0.28
HLA‐B*27 and/or B*57	2.68 (1.51 to 4.76)	0.001	3.96 (1.93 to 8.13)	<0.001
HIV‐RNA load at inclusion^a^	0.25 (0.16 to 0.40)	<0.001	0.31 (0.19 to 0.52)	<0.001
HIV‐DNA load at inclusion^a^	0.12 (0.06 to 0.27)	<0.001	0.19 (0.09 to 0.44)	<0.001
Positive HCV serology	0.51 (0.20 to 0.31)	0.16	0.82 (0.25 to 2.62)	0.73

HLA, human leucocyte antigen; HCV, hepatitis C virus; IQR, interquartile range; ORs, Odds ratios.

^a^ORs calculated for a 1‐log_10_ increase.

## 4 Discussion

The question of the evolution of HIV reservoirs over the course of long‐term control, which is characteristic of HIC, may offer new insights into the mechanisms of HIV persistence in HIC. The ANRS CODEX cohort is a large cohort with long‐term follow‐up, and it provides the opportunity to study HIV reservoirs, owing to the regular sampling of blood collected over years. This marker is not perfect as it includes both integrated and unintegrated forms and could overestimate the reservoir size. However, the role of the defective forms is associated with viral proteins production, resulting in immune activation, which participates to the pathogenesis and maintenance of HIV reservoirs 12, 15, 16. The predictive value of total HIV‐DNA level on the course of infection indicates that it is clinically relevant 12, 15, 16 even if it quantifies all HIV‐DNA forms, including infectious and defective viruses 17, 18. It is then a convenient marker to monitor the reservoir size in such a large series of HIV‐infected patients with frozen samples. Moreover, this assay has a greater precision and reproducibility in the context of a low level of detection than is possible for other markers of HIV reservoirs 12, 15, 16. Indeed, this marker as well as HIV‐RNA must be quantified by ultrasensitive assays, which are needed in the context of control in HIC.

In this large cohort of 202 HIC, we confirmed that HIV blood reservoirs are low; in fact, the reservoirs in HIC are much lower than those in adults and children who have received combined antiretroviral treatment for several years 1, 12, 19. Nevertheless, various levels of HIV‐DNA were observed in HIC, including among individuals with HLA‐B*27 and/or B*57 alleles. We identified a group of HIC with particularly low HIV‐DNA levels. The different parameters associated with these low levels of HIV‐DNA confirmed a unique mechanism of control that is very efficient in those “super” HIC. Conversely, higher HIV‐DNA levels were associated with higher frequencies of activated CD4+ T cells, which was in accordance with previous data 20. IP‐10 is a pro‐inflammatory chemokine that is positively correlated with the expression of activation markers in CD8 and CD4 T cells 21 and is lower in HIC who maintain CD4 T‐cell counts >500 cells/mm^3^ for more than seven years after HIV‐1 diagnosis 22. IP‐10 levels were positively correlated with levels of blood reservoirs in the present study. Higher HIV‐DNA levels were also associated with a higher HIV‐suppressive capacity mediated by CD8 T cells. This completes our previous data demonstrating that HIV controllers with higher levels of reactivable viruses had higher levels of CD8 T‐cell responses 23. These results indicate that a strong HIV‐specific CD8+ T‐cell response during the chronic phase of control is maintained in those HIV controllers with detectable levels of viral replication. When the control is strong enough, the systemic CD8 T‐cell response (and activation) would decrease because most replication‐competent viruses would be cleared by the efficient immune responses, or unable to replicate due to intracellular mechanisms of viral restriction 24, and periodic reactivation can be locally controlled by immune responses. Lastly, the higher HIV‐DNA loads observed in HIC seropositive for HCV could be linked to their higher levels of activation and inflammation. Moreover, positive HCV serology has been associated with a higher risk of progression in HIC 3, 25.

Interestingly, we report for the first time in such a large cohort with long‐term follow‐up that the dynamics of HIV reservoirs vary according to different groups among HIC. Individuals with higher levels of viral replication during the follow‐up experienced an increase in HIV reservoir size, and this can be linked to the higher risk of progression that has been previously described in such patients 25. Interestingly, we report a decrease in HIV‐DNA level over the course of years in half of HIC, suggesting a progressive loss of infected cells. Interestingly, a decrease in HIV reservoir size over time and a low level of contribution of long half‐life T cells to this reservoir were also observed in post‐treatment controllers who lacked protective HLA alleles 26. The negative slope observed in HIC is similar to that observed for patients after 32 months of combined antiretroviral therapy initiated as soon as the primary infection (−0.032 log/year) 27. This is in contrast with the significant HIV DNA increase that we described using the same assay, during the natural history of HIV infection in an historical cohort of non‐controller patients (before the cART era). The median baseline HIV DNA load in these non‐controller patients was much higher than for HIV controllers (1250 copies/million PBMC). With the same mathematical models, we described an increase of +0.105 log copies/million PBMCs/year for rapid progressors, who developed AIDS during follow‐up, (n = 34, 111 samples; +5332 copies/million PBMC over six years) and +0.096 log copies/million PBMCs/year for slower progressors, who did not reach the AIDS stage during the follow‐up time (n = 63, 229 samples; +4709 copies/million PBMC over six years) 28.

The decrease in HIV‐DNA is notably observed in HIC with the protective HLA‐B*27 and/or B*57 alleles, which are linked to efficient responses against HIV 29. This decrease in HIV‐DNA could then be one of the mechanisms underlying the association of this allele with protection against HIV‐1 disease progression in controllers, as has been recently described 22. HLA‐B*57 has been previously associated with the restriction of viral replication in long‐term non‐progressors (LTNPs) 30, 31. Moreover, we previously reported that LTNPs with HLA‐B*27/B*57 had a lower infection level of central memory CD4 T cells than other LTNPs 32. Central memory CD4 T cells are characterized by long half‐lives and high proliferative capacities, and they play a major role in immune responses. In LTNPs with HLA‐B*27 and/or B*57 alleles, central memory CD4 T‐cell protection was correlated with the preservation of central memory CD4 T‐cell counts, which correlated positively with the magnitude of HIV Gag‐specific CD8 T cells 32. The fact that cells with short half‐lives contribute substantially to the HIV reservoirs in those individuals with HLA‐B*27/B*57 alleles could help account for the decrease in HIV reservoir size in these HIC, because cells with short half‐lives can be more easily eliminated than long‐lived central memory CD4 T cells. HIC with HLA‐B*27 and/or B*57 alleles have a tighter control of infection and as a consequence they may more efficiently eliminate infected cells.

Given that low levels of HIV‐RNA, low levels of HIV‐DNA and the presence of HLA‐B*27 and/or B*57 alleles are significantly and independently associated with a decrease in HIV reservoir size in these HIC, this decrease could be linked to several mechanisms, including a relative intrinsic resistance of CD4 T cells/macrophages to infection, as previously described 24; a low activation level of CD4 T cells that limits the number of potential target cells for new infection; a low replenishment by infection of new cells due to low residual replication which can persist in natural controllers as previously described 33, 34, 35; and a low level of proliferation of infected cells because of the protection of long‐lived T cells against HIV infection and a short half‐life of HIV‐infected cells for the same reasons.

Another non‐exclusive hypothesis to explain the decrease in infected cells over time could be linked to the composition of total HIV‐DNA. The total HIV‐DNA quantified in this study thus includes all forms of HIV‐DNA, including integrated HIV‐DNA, the main form of HIV persistence, as well as episomal and linear unintegrated forms. Graf *et al*. described that elite suppressors had large excess amounts of 2‐LTR HIV‐DNA among the total HIV‐DNA in their blood 11. Unlike the integrated forms, these forms are diluted during cell division and can be progressively eliminated over time.

We hypothesize that the decrease in HIV‐DNA contributes to the maintenance of a high degree of control, and vice versa, and can partly explain the clinical, immunologic and viral characteristics of HIC. These findings reinforce the fact that there is a well‐balanced group of HIC with a very low risk of progression. HIC, a rare group identified among the HIV‐positive population when they have several years of control, may not all need to initiate an antiretroviral treatment. A personalized management and precision medicine could be beneficial for optimizing the clinical care of these individuals.

## 5 Conclusions

To conclude, among HIC, half of individuals present a very high level of control of the infection, with a slow and progressive decrease in the HIV blood reservoir and a very low level of viral replication. As HIC have been proposed as a model for remission, this subgroup with a very high degree of control may represent the most informative patient population in this regard. Some factors are associated with this unique level of control, namely, very low levels of HIV replication, very low total HIV‐DNA levels and the presence of protective alleles.

## Competing interests

The authors declare to have no conflict of interest.

## Authors’ contributions

VAF, FB, OL and CR designed the research study. VAF, TB, EG, FB, PL, CL, NN, PT, VM and ASC performed the research. VAF, TB, FB, ASC, BA, LM, OL and CR analysed the data. VAF and CR wrote the paper. TB, FB, ASC, BA and OL critically revised the manuscript. All authors approved the manuscript.

## Appendix 1

### The ANRS CODEX‐CO21 cohort study group

Dr Jean‐Pierre Faller, Mme Patricia Eglinger, Service des Maladies Infectieuses, CH de Belfort‐Montbéliard, Belfort. Pr Pascal Roblot, M David Plainchamp, Service de Médecine Interne, CHU Poitiers‐La Milétrie, Poitiers. Dr Hugues Aumaître, Mme Martine Malet, Service des Maladies Infectieuse et Tropicales, CH de Perpignan, Perpignan. Dr Christine Rouger, Pr Gérard Rémy, Melle Kmiec Isabelle, Service des Maladies Infectieuses, CHU Reims‐Hôpital Robert Debré, Reims. Dr Jean‐Luc Delassus, Service de Médecine Interne, CHI Ballanger, Aulnay Sous‐Bois. Dr Alain Devidas, Service d'Hématologie, CH Sud‐Francilien ‐Hôpital Gilles de Corbeil, Corbeil ‐ Evry. Dr Eric Froguel, Mme Sylvie Tassi, Service de Médecine Interne‐Maladies Infectieuses, CH de Marne la Vallée, Jossigny. Dr Philippe Genet, Mme Juliette Gerbe, Service Hématologie‐Immunologie, Centre Hospitalier Victor Dupouy, Argenteuil. Pr Olivier Patey, Mr Richier Laurent, Service des Maladies Infectieuses et Tropicales, CHI Villeneuve Saint Georges, Villeneuve Saint Georges. Dr Marie‐Christine Drobacheff, Dr Aurélie Proust, Service de Dermatologie, Hôpital Saint‐Jacques, Besançon. Dr Helder Gil, Service de Maladies Infectieuses et Tropicales, Besançon. Dr Laurence Gérard, Pr Eric Oksenhendler, Service d'Immuno‐pathologie, Hôpital Saint Louis, Paris. Pr Frédéric Lucht, Mme Véronique Ronat, Service de Maladies Infectieuses, Hôpital Bellevue, Saint Etienne. Pr Michel Dupon, Dr Hervé Dutronc, Mme Séverine Le Puil, Service des Maladies Infectieuses, CHU‐ Hôpital Pellegrin, Bordeaux. Pr Jean‐Luc Schmit, Mme Nathalie Decaux, Service de Pathologies Infectieuses, CHU‐ Hôpital Nord, Amiens. Pr Jean‐Michel Molina, Dr Caroline Lascoux, Mme Sylvie Parlier, Service de Maladies Infectieuses et Tropicales, Hôpital Saint Louis, Paris. Dr Jean‐Pierre Bru, Mme Gaëlle Clavere, Service des Maladies Infectieuses, Centre Hospitalier Annecy, Annecy. Pr Olivier Lambotte, Pr Jean‐François Delfraissy, Pr Cécile Goujard, Mme Katia Bourdic, Service de Médecine Interne, Hôpital de Bicêtre, Le Kremlin Bicêtre. Pr Jean‐François Bergmann, Mme Maguy Parrinello, Service de Médecine Interne A, Hôpital Lariboisière, Paris. Dr Gilles Pichancourt, Service Hématologie, Hôpital Henri Duffaut, Avignon. Dr Yves Welker, Service de maladies Infectieuses, CHI de Poissy‐Saint Germain en Laye, Saint Germain en Lay. Dr Alain Lafeuillade, Mme Philip Gisèle, Service d'Infectiologie, CHITS Hopital Sainte Musse, Toulon. Pr Christophe Rapp, Melle Lerondel, Service des Maladies Infectieuses, Hôpital d'Instruction des Armées Bégin, Saint Mandé. Dr Pierre de Truchis, Mme Huguette Berthe, Département de Médecine Aigue Spécialisée, Hôpital Raymond Poincarré, Garches. Dr Vincent Jeantils, Mme Fatouma Mchangama, Unité de Maladies Infectieuses, Hôpital Jean Verdier, Bondy. Pr. Daniel Vittecoq, Mme Claudine Bolliot, Service des Maladies Infectieuses, Hôpital de Bicêtre, Le Kremlin Bicêtre. Dr Paul Henri Consigny, Mme Fatima Touam, Consultation de Maladies Infectieuses, Centre Médical de l'Institut Pasteur, Paris. Pr Gilles Pialoux, Mme Sophie le Nagat, Service des Maladies Infectieuses, Hôpital Tenon, Paris. Pr Olivier Bouchaud, Mme Patricia Honoré, Service de Médecine Interne et Endocrinologie, Hôpital Avicenne, Bobigny. Pr François Boué, Mme Mariem Raho‐Moussa, Service de Médecine Interne, Hôpital Antoine Béclère, Clamart. Pr Laurence Weiss, Dr Lio Collias, Service d'Immunologie Clinique, HEGP, Paris. Pr Dominique Salmon‐Céron, Mme Marie‐Pierre Pietri, Service de Médecine Interne et centre références Maladies Rares, Hôpital Cochin, Paris. Dr Zucman, Pr Olivier Blétry, Mme Dominique Bornarel, Service de Médecine Interne, Hôpital Foch, Suresnes. Dr Emmanuel Mortier, Mme Zeng Feng, Service de Médecine Interne, Hôpital Louis Mourier, Colombes. Pr Jean‐Daniel Lelièvre, Service d'Immunologie Clinique, Hôpital Henri Mondor, Créteil. Pr Christine Katlama, Mme Yasmine Dudoit, Service des Maladies Infectieuses, Hôpital Pitié‐Salpêtrière, Paris. Dr Anne Simon, Mme Catherine Lupin, Service des Maladies Infectieuses, Hôpital Pitié‐Salpêtrière, Paris. Pr Pierre‐Marie Girard, Mme Michèle Pauchard, Service des Maladies Infectieuses, Hôpital Saint Antoine, Paris. Dr Sylvie Abel, Dr André Cabié, Service de Maladies Infectieuses et Tropicales, Hôpital Pierre Zobda‐Quitman, Fort de France, Martinique. Dr Pascale Fialaire, Dr Jean‐Marie Chennebault, M Sami Rehaiem, Service des Maladies Infectieuses et Tropicales, CHU Angers, Angers. Dr Luc de Saint Martin, Dr Perfezou, M Jean‐Charles Duthe, Service de Pneumologie, CHU de Brest, Brest. Pr Philippe Morlat, Mme Sabrina Caldato, Service de Médecine Interne, CHU‐ Hôpital Saint André, Bordeaux. Pr Didier Neau, Mme Séverine LE Puil, Service des Maladies Infectieuses A, CHU‐ Hôpital Pellegrin, Bordeaux. Pr Pierre Weinbreck, Dr Claire Genet, Service des Maladies Infectieuses, CHU de Limoges, Limoges. Dr Djamila Makhloufi, Mme Florence Garnier, Service d'Immunologie Clinique, HCL‐ Hôpital Edouard Herriot, Lyon. Dr Isabelle Poizot‐Martin, Dr Olivia Fauche, Mme Alena Ivanova, Service Hématologie‐ Cisih, Hôpital Sainte Marguerite, Marseille. Pr Patrick Yeni, Dr Sophie Matheron, Mme Godard Cyndi, Service des Maladies Infectieuses, Hôpital Bichat Claude Bernard, Paris. Pr François Raffi, Mr Hervé Hüe, Service de Médecine Interne, Hôpital de l'Hôtel Dieu, Nantes. Dr Philippe Perré, Service de Médecine Interne post‐Urgence, Centre Hospitalier Départemental, La Roche sur Yon. Pr Pierre Marie Roger, Mme Aline Joulie, Service des Maladies Infectieuses, CHU‐ Hôpital l'Archet, Nice. Pr Éric Rosenthal, Service Médecine Interne, CHU‐ Hôpital l'Archet, Nice. Pr Christian Michelet, Dr Faouzi Souala, Mme Maja Ratajczak, Service des Maladies Infectieuses, CHU‐Hôpital Pontchaillou, Rennes. Dr Marialuisa Partisani, Mme Patricia Fischer, HUS‐Hôpital Civil, Strasbourg. Pr Louis Bernard, Mme Pascale Nau, Service des Maladies Infectieuses, CHRU‐ Hôpital Bretonneau, Tours. Pr Bruno Marchou, Mme Florence Balsarin, Service des Maladies Infectieuses, CHU‐Hôpital Purpan, Toulouse. Pr Renaud Verdon, Mr Philippe Feret, Service des Maladies Infectieuses, CHU‐ Hôpital de la Côte de Nacre, Caen. Dr Christine Jacomet, Service des maladies Infectieuse, CHU Gabriel Montpied, Clermont Ferrand. Dr Lionel Piroth, Mme Sandrine Gohier, Service de Maladies Infectieuses et Tropicales, CHU‐Hôpital du Bocage, Dijon. Dr Pascale Leclercq, Mme Gerberon, Service Médecin Aigue spécialisée, CHU‐Hôpital Albert Michallon, Grenoble. Dr Agnés Meybeck, Dr Raphaël Biekre, Service des Maladies Infectieuses, CH‐ Hôpital Gustave Dron, Tourcoing. Pr Thierry May, Mme Bouillon, Service de Maladies Infectieuses et Tropicales, CHU Nancy, Nancy. Pr François Caron, Dr Yasmine Debab, M David Theron, Service de Maladies Infectieuses et Tropicales, CHU‐ Hôpital Charles Nicolle, Rouen. Dr Patrick Miailhes, M Stanislas Ogoudjobi, Service de Maladies Infectieuses et Tropicales, HCL‐ Hôpital de la Croix Rousse, Lyon. Pr Patrick Mercié, Service de Maladies Infectieuses et Tropicales, CHU‐ Hôpital Saint André, Bordeaux. Dr Marc Gatfosse, Service de Médecine Interne, CH René Arbeltier, Coulommiers. Dr Martin Martinot, Mme Anne Pachart, Service de Maladies Infectieuses‐Médecine Interne, Hôpitaux Civils de Colmar, Colmar. Dr Patrice Poubeau, Service de Pneumo‐phtisiologie, Centre Hospitalier Sud Réunion ‐ Hôpital de St Pierre, Saint Pierre, La Réunion. Dr Agnès Uludag, Service de Médecine Interne, Hôpital Beaujon, Clichy. Dr Philippe Arsac, Mme Lydia Bouaraba, Service de Médecine Interne, CHR Orléans‐ Hôpital Porte Madeleine, Orléans. Dr Isabelle De Lacroix Szmania, M Laurent Richier, Service de Médecine Interne, Centre Hospitalier Intercommunal, Créteil. Dr Vincent Daneluzzi, Service de Médecine A, CASH ‐ Hôpital Max Fourestier, Nanterre. Dr Elisabeth Rouveix, Service de Médecine Interne 2, Hôpital Ambroise Paré, Boulogne. Dr Geneviève Beck‐Wirth, Service d'Hématologie Clinique VIH, Centre Hospitalier de Mulhouse, Mulhouse. Dr Philippe Romand, Service de Pneumologie, CHI Les Hôpitaux du Léman, Thonon les Bains. Dr Laurent Blum, Mme Martine Deschaud, Service Médecine‐Gastroentérologie, Centre hospitalier René Dubos, Pontoise. Dr Christophe Michau, Service de Médecine Interne, Centre Hospitalier de Saint Nazaire, Saint Nazaire. Dr Christian Bernard, Mme Florence Salaun, Service de Médecine Interne, CHR Metz Thionville Hôpital Notre Dame de Bon Secours, Metz. Dr Philippe Muller, Service de Dermatologie, Hôpital Beauregard, Thionville. Dr Yves Poinsignon, Service de Médecine Interne, Hôpital Prosper Chubert, CHBA, Vannes. Dr Annie Lepretre, Mme Martine Deschaud, Service de Médecine Interne, Hôpital Simone Veil, Eaubonne. Dr Thierry Lambert, Consultation d'Hématologie, CHU de Bicêtre, Le Kremlin Bicêtre. Dr Laurent Hocqueloux, Mme Barbara de Dieulevault, Service de Maladies Infectieuses et Tropicales, Hôpital Orléans la Source, Orléans. Dr Patrick Philibert, Mme Mame Penda Sow, Consultation de Médecine Interne, Hôpital Européen Marseille, Marseille. Pr Albert Sotto, Mme Doncesco, Service des Maladies Infectieuses et Tropicales, CHU Caremeau, Nîmes. Pr Jean‐Paul Viard, Mme Agnés Cros, Centre de diagnostic et de thérapeutique, Hôpital Hotel Dieu, Paris. Dr Marc De Lavaissiere, Service Médecine Interne, CHG de Montauban, Montauban. Dr Pascale Perfezou, M Jean Charles Duthe, Service de Pneumologie, CH de Cornouaille‐Hôpital Laennec, Quimper. Dr Catherine Gaud, Service Immunologie Clinique, Centre Hospitalier Félix Guyon, Ile de la Réunion. Dr Mathilde Aurore Niault, Mme Virginie Mouton‐Rioux, Service d'hématologie, Maladies Infectieuses, CH Bretagne Sud, Lorient. Dr Jean–Philippe Talarmin, M Jean Charles Duthé, Service Médecine Interne, CH de Cornouaille‐Hôpital Laennec, Quimper. Dr Dupont Mathilde, M Stéphane Natur, Service des Maladies Infectieuses et Tropicales, CH Saint Malo, Saint Malo. Dr Hikombo Hitoto, M Ali Mahamadou Ibrahim, Service de Maladies Infectieuses et Tropicales, Centre Hospitalier Le Mans, Le Mans.
